# Working Memory Training Using Mental Calculation Impacts Regional Gray Matter of the Frontal and Parietal Regions

**DOI:** 10.1371/journal.pone.0023175

**Published:** 2011-08-23

**Authors:** Hikaru Takeuchi, Yasuyuki Taki, Yuko Sassa, Hiroshi Hashizume, Atsushi Sekiguchi, Ai Fukushima, Ryuta Kawashima

**Affiliations:** 1 Smart Ageing International Research Center, Institute of Development, Aging and Cancer, Tohoku University, Sendai, Japan; 2 Division of Developmental Cognitive Neuroscience, Institute of Development, Aging and Cancer, Tohoku University, Sendai, Japan; 3 Department of Functional Brain Imaging, Institute of Development, Aging and Cancer, Tohoku University, Sendai, Japan; Université Pierre et Marie Curie, France

## Abstract

Training working memory (WM) improves performance on untrained cognitive tasks and alters functional activity. However, WM training's effects on gray matter morphology and a wide range of cognitive tasks are still unknown. We investigated this issue using voxel-based morphometry (VBM), various psychological measures, such as non-trained WM tasks and a creativity task, and intensive adaptive training of WM using mental calculations (IATWMMC), all of which are typical WM tasks. IATWMMC was associated with reduced regional gray matter volume in the bilateral fronto-parietal regions and the left superior temporal gyrus. It improved verbal letter span and complex arithmetic ability, but deteriorated creativity. These results confirm the training-induced plasticity in psychological mechanisms and the plasticity of gray matter structures in regions that have been assumed to be under strong genetic control.

## Introduction

Working memory (WM) is the limited capacity storage system involved in the maintenance and manipulation of information over short periods of time [1]. Individual working memory capacity (WMC) is correlated with a wide range of cognitive functions [1]. On the other hand, WMC and creativity show a lot of opposing psychological, pathological, pharmacological and genetic characteristics (for detail, see [2]). Previous neuroimaging studies using diverse imaging methods have investigated the neural correlates of WM and WMC [1].

Previous studies have shown WM training's effect on psychological measures and neural systems. It has been shown that training on cognitive tasks, including WM tasks, can improve performance on trained tasks as well as on some untrained transfer tasks such as memory tasks, intelligence, and response inhibition tasks [3], [4], [5], [6], [7], [8]. Also, while lateral prefrontal and parietal regions play a key role in WM [8], altered patterns of brain activity during the untrained cognitive tasks, altered density of cortical dopamine D1 receptors, and altered white matter integrity after training on WM tasks that are associated with prefrontal and parietal regions have been demonstrated [4], [5], [8], [9], [10]. Nevertheless, no previous study has observed the effect of WM task training on gray matter (GM) structures nor diverse cognitive functions such as spatial abilities and creativity. Considering individual working memory capacity (WMC) is correlated with a wide range of cognitive functions [1], how the training of WM is associated with changes of those cognitive function is a matter of interest. Furthermore, previous neuroimaging studies that investigated the effects of WM task training did not have appropriate control groups with placebo training.

In this study, we focused on these unresolved issues using newly developed computer-based mental calculation task training, which requires manipulation of maintained information and is often referred to as typical of WM tasks. Using various psychological measures such as non-trained WM tasks and a creativity task, along with voxel-based morphometry (VBM) [11], we investigated the effects of training on WM tasks using mental calculation. VBM has been widely used as a tool to investigate the structural change following interventions at the whole brain level including subcortical structures [12], [13] and it yields very consistent **results** with other voxel-based structural method as well as an ROI analysis [14], [15], [16]. We hypothesized regional gray matter structures in the lateral PFC and possibly parietal regions are affected by the training. However, given the previous training studies have shown training related increase, decrease and nonlinear changes (decrease after transient increase) of regional gray matter structures, we did not expect the direction of the change [17], [18], [19], [20].

Subjects were divided into three groups: a group with intensive adaptive training of WM using mental calculation (IATWMMC), a placebo group with non-adaptive training of WM using mental calculation (placebo intervention) and a group with no training at all. The subjects in the IATWMMC group performed mental multiplication and mental addition tasks using a computer program in which the task difficulty was modulated to adapt to the subjects' performances. As reviewed in Takeuchi et al. [8], various WM training tasks were used in the WM training studies, such as basic types of WM tasks such as digit span, updating WM tasks such as N-back task, complex WM tasks in which subjects must remember the presented stimuli and perform other processing tasks during or between the presentation of stimuli and so on. In this study, we used IATWMMC because, mental calculation is often referred as typical of WM tasks and these tasks required strong manipulation of maintained information. Before and after the five-day intervention, each subject participated in both MRI experiments and psychological experiments during which they went through cognitive measures.

## Methods

### Ethics statement

In accordance with the Declaration of Helsinki (1991), Written informed consent was obtained from each subject. This study was approved by the Ethics Committee of Tohoku University.

### Participants

Fifty-five healthy, right-handed individuals (42 men and 13 women) participated in this study. The mean age was 21.7 years (standard deviation [SD], 1.4). All subjects were university students or postgraduate students. Females were included in this study as was the case with almost all of the intervention studies of this kind. All subjects had normal vision, none had a history of neurological or psychiatric illness, and none reported any recent use of psychoactive drugs or antipsychotic drugs. A history of psychiatric illnesses or recent drug use was assessed with our laboratory's routine questionnaire in which each subject answered questions about whether they had or have any of a list of illnesses and also listed drugs they had taken recently. Handedness was evaluated using the Edinburgh Handedness Inventory [21]. Written informed consent was obtained from each subject. This study was approved by the Ethics Committee of Tohoku University. Several participants participated in the pre- and post- MRI and psychological experiments at the same time. Participants were randomly assigned to either the intervention group (the IATWMMC or the placebo group) or to the no-intervention group. But participants in the same intervention group period were all assigned to the same training protocol group (the IATWMMC group or the placebo group). This means for example, participants in the period of January 7^th^–January 14^th^ are assigned to the IATWMMC group when they are assigned to the intervention group, but participants in the period of January 21^st^–January 28^th^ are assigned to the placebo group when they are assigned to the intervention group. In addition, participants chose the periods in which they wanted to participate by themselves. None of the participants were notified that there were three groups (rather than just the intervention group and the no-intervention group) until after the post MRI and psychological experiments. In other words, participants in the placebo controlled group did not know they were practicing placebo training tasks until the end of the experiment. The IATWMMC group consisted of 18 participants (13 men and five women) and the mean age of the IATWMMC group was 21.9 years (standard deviation [SD], 1.5). The placebo group consisted of 18 participants (twelve men and six women) and the mean age of the placebo group was 21.6 years (standard deviation [SD], 1.6). The no-intervention group consisted of 19 participants (17 men and two women) and the mean age of the no-intervention group was 21.7 years (standard deviation [SD], 1.3). The IATWMMC and placebo intervention and no-intervention groups did not differ significantly (P>0.2, ANOVA) in basic background characteristic such as age, sex, and the score of Raven's Advanced Progressive Matrix [22], which measures cognitive ability that is central to general intelligence [23]. One participant in the IATWMMC group and one participant in the placebo intervention group terminated their training prior to completion. Furthermore, another participant in the IATWMMC group repeated intentional mistakes during the training. These three subjects were excluded from the further analysis of the effects of the training.

### Procedure

The experimental and placebo training programs were computerized, in-house developed Borland C++ programs that consisted of mental multiplication tasks and mental addition tasks. Participants in the experimental and placebo training groups undertook five days of training within six days. Training each day lasted about four hours which usually included two 10-minuite breaks. All participants were MRI scanned and took psychological tests immediately before and after this six-day period. This means the participants were MRI scanned and took psychological tests in one day and one week after that. The no-intervention group did not receive any training or perform any specific activity during the period separating the two MRI sessions.

### Training tasks

The mental multiplication task in the IATWMMC is an adaptive training of mental multiplication calculations. The program for the mental multiplication task in the IATWMMC group is designed to assist participants' mental calculation abilities by allowing subjects to check whether the intermediate result of their mental multiplication is correct. The “intermediate result” of mental multiplication refers to the answer in each column when multiplication problems are solved in the following way: when the problem is 37×45, the intermediate result of the first column is 37×5 = 185, and that of the second column is 37×4 = 148). Subjects are asked to solve mental multiplication problems in a normal way (as Japanese or English speakers do computations on paper (see [24]) in their minds and not to solve problems in any other way. Subjects must continue the task until they get the correct answer. After they get the final correct answer, they are asked to give the intermediate answers to the problem without looking at the problem in order to rule out the use of any other possible strategies that do not solve the problems in a normal way. Giving up on or experiencing too many failures during the calculation, or running out of time (one hour), are conditions considered to be failures. There is a 1 hour time limit in this task, which means there is no virtually no time limit in this task. This measure was taken so that the time (not the subjects' abilities) did not prevent subjects from reaching the solution. If participants answer correctly, the problems become more difficult (the task starts from two-digit times two-digit multiplication and then becomes two-digit times three-digit multiplication and then three-digit times three-digit multiplication and then three-digit times four-digit mental multiplication). Two failures in a row make the problems less difficult. The computerized task for mental addition is programmed for the intensive adaptive or progressive training of mental addition calculations. Ten two-digit numbers are presented one by one and the subjects are asked to add them. If they get the correct answer, the interstimulus interval (ISI) becomes shorter [ISI becomes (original ISI)×(0.9)×(0.9)×(0.9)]. Five wrong answers in a row makes the problems less difficult [ISI becomes (original ISI)×(10/9)]. The two trainings were interleaved. The subjects in the placebo intervention group perform similar tasks, except the difficulty of the tasks doesn't change from the initial points (two-digit times two-digit multiplication in the mental multiplication task, ten-seconds-ISI in the mental addition task). In this placebo mental multiplication task, making three mistakes in one problem is considered to be a failure and the next problem appears. The IATWMMC group's subjects know the level of the tasks they are doing and the placebo intervention group's subjects receive feedback on their current performance (accuracy) in every training session during the training. Thus, subjects of the placebo training go through the same amount of training as the IATWMMC group's subjects and receive feedback on their task performance. WM training without intensive adaptive training does not cause an increase in general WM capacity [25]. After the experiment, the individuals complete a questionnaire to ascertain each subject's subjective feelings about the effects of the training, the subjective fatigue that subjects felt during the task [which was measured by a visual analogue scale (VAS)], the strategies that they used while performing the training task, and so on.

### Psychological outcome measures

For pre- and post- training evaluation, a battery of neuropsychological tests and questionnaires was administered. This battery includes the following contents. [A] Raven's Advanced Progressive Matrices [RAPM; 22], a nonverbal reasoning task; [B] the arithmetic task in the WAIS-III [26], a WM task using mental calculation; [C] the digit symbol task in the WAIS- III [26], a processing speed task; [D] the Stroop task (Hakoda's version) [27], which measures response inhibition and impulsivity. This version of the Stroop task is a matching-type requiring subjects to choose and check correct answers, unlike the traditional oral naming task. The test consists of two control tasks, a Stroop task and a reverse-Stroop task. Two independent measures, reverse-Stroop interference rate and Stroop interference rate, are calculated. [E] The S-A creativity test [28], which measures creativity. A detailed discussion of the psychometric properties of this instrument and how it was developed is found in the technical manual of this test [28]. The test is used to evaluate creativity through divergent thinking [28] and it involves three types of tasks. The first task requires subjects to generate unique ways of using typical objects. The second task requires subjects to imagine desirable functions in ordinary objects. The third task requires subjects to imagine the consequences of ‘unimaginable things’ happening. The S-A test scores the four dimensions of the creative process (fluency, originality, elaboration, and flexibility). In this study, the sum of the graded scores of the four dimensions was used in the analysis. For more details including the psychometric properties of this test, sample answers to the questionnaire, and the manner in which they were scored, see our previous works [29], [30]. [F] Arithmetic tasks, which are similar to the ones constructed by Grabner et al. [31]. These tests measure multiplication performance consisting of two forms of one-digit times one-digit multiplication problems (a simple arithmetic task with numbers between 2 and 9) and two forms of two-digit times two-digit multiplication problems (a complex arithmetic task with numbers between 11 and 19) . The two forms of each task are the same, but the numbers used in the problems are different. Each form of the simple arithmetic task is presented with a time limit of 30 s and each form of the complex arithmetic task is presented with time limits of 60 s. [G] Letter mental rotation task, in which a pair of Japanese letters (one, a normal word and the other, either a rotated normal letter or a rotated mirrored image), are presented and participants are asked to judge whether the two presented letters would be the same or not after they are rotated. [H] The letter span task, a verbal WM task. This test is administered like the Digit span task [26], except that instead of digits, Japanese letters are used. This measure was taken to rule out the possibility that the expected improvement in the span task following our training resulted because participants became habituated to remembering numbers. [I] Trail making tests A and B, which measures cognitive flexibility [32]. No other cognitive tests were used in this study. Questionnaires that were designed to assess mainly the traits of subjects were collected from the subjects but not described in this study because they were apparently not designed to assess the effects of a five-day intervention. Except self-report questionnaires, all neuropsychological assessments were performed by postgraduate and undergraduate students who were kept blind to the group membership of participants.

### Image acquisition

All MRI data acquisition was conducted with a 3-T Philips Intera Achieva scanner. Using a MPRAGE sequence, high-resolution T1-weighted structural images (240×240 matrix, TR = 6.5 ms, TE = 3 ms, FOV = 24 cm, 162 slices, 1.0 mm slice thickness) were collected. In this study, only these T1-weighted structural images were analyzed. The diffusion-weighted data were acquired only in the pre- MRI experiment by using a spin-echo EPI sequence. Arterial spin labeling images were obtained only in the pre- MRI experiment. All the participants are assigned to our on-going study to investigate the association among brain images, cognitive functions, and their age-related changes. The images that were taken in the pre- MRI experiment were used in our previous study [30] and are going to be used in our future study, but not in this study. Furthermore, functional MRI data were obtained while the subjects were performing the N-back task and mental calculation task in the pre- MRI and post- MRI scans, but functional MRI data were not analyzed in this study. The details of parameters in these scans were not described in this study, since these scans were not used in this study. However, for the details of diffusion-weighted data, see our previous work [30].

### VBM analysis

Data pre-processing of the morphological data was performed with VBM2 software [33], an extension of SPM2. Default parameter settings were used [33]. In order to reduce the scanner-specific bias, we created a customized GM anatomical template from the pre-intervention data of all the participants in this study. To facilitate optimal segmentation, we estimated normalization parameters using an optimized protocol [11]. In addition, we performed a correction for volume changes (modulation) by modulating each voxel with the Jacobian determinants derived from spatial normalization, allowing us to also test for regional differences in the absolute amount of GM [34]. Subsequently, all images were smoothed by convolving them with an isotropic Gaussian Kernel of 10 mm full-width at half maximum. Finally, the signal change in regional gray matter volume (rGMV) between pre- and post- intervention images was computed at each voxel for each participant. In this computation, we included only voxels that showed GMV values>0.10 in both pre- and post- scans to avoid possible partial volume effects around the borders between GM and WM as well as between GM and CSF. The resulting maps representing the rGMV change between the pre- and post- MRI experiments (rGMV post – rGMV pre) were then forwarded to the group level analysis, described in the next section.

Finally, to ensure that there were no potential differences between the groups in the amount of rGMV and that training related changes cannot be explained by pre-training differences in gray matter, we compared pre-training rGMV between the IATWMMC group and the combined control groups. We used the ANCOVA option of SPM5 for this analysis with no covariates (which is equal to ANOVA). In the group analysis, we included only voxels that showed a GM value>0.10 to avoid the possibility of partial volume effects.

### Statistics in group level analysis in imaging and behavioral data

The behavioral data were analyzed using the statistic software SPSS 16.0 (SPSS Inc., Chicago, IL). Since our primary interest is only the superiority (or beneficial effects) of the intervention training, in our behavioral analysis test-retest changes in the group of interest were compared to the test-retest changes in the control group using one-tailed tests (*P*<0.05) as was performed in previous studies [3], [35]. However, two-tailed tests were used in behavioral measures in which the definition of ‘superiority’ was not clear; namely, for the inverse Stroop interference rate which shows age related decline [36] and for an increase in the Stroop interference rate in patients with schizophrenia [37]. The two-tailed tests were also used to compare group differences in the changes in the creativity test scores, which are associated with impaired selective attention systems, psychosis and cognitive disinhibition [38], [39], [40]. Creativity seems as an obvious positive trait, however there are tremendous amount of literatures that show creativity is associated with psychopathologies and impaired selective attention system (for the full discussion of this matter, see [2]). Especially, WMC and creativity show a lot of opposing psychological, pathological, pharmacological and genetic characteristics (for detail, see [2]). For example, the prevalent genotype that is associated with lower WMC [41] is associated with increased creativity [42]. On the other hand, Ritalin (methylphenidate) administration significantly decreased symptoms of attention deficit hyperactive disorder and creativity [43] while improving WMC [44]. In the psychological and morphological analyses, first the placebo group was compared with the no-intervention group using one-way analyses of covariance (ANCOVAs) with the difference between pre- and post- test measures as dependent variables and pretest scores as covariates [in the VBM analyses, total gray matter volume in the pre-measurement, pretest scores of general intelligence (RAPM) measures, and two measures of WM (the arithmetic task of WAIS-III and the letter span task), were used as covariates] to exclude the possibility that any pre-existing difference of measurement between the groups affected the result of each measure. Complex arithmetic ability was not included as a covariate because it is relatively little to do with WM compared with the arithmetic task of WAIS-III (note the latter is a typical WM task in that subjects remember told complex information and do mental calculation based on the information, even though the task is called “arithmetic”, while the former is not the case and the two tasks are very much different in relationship with WM). In this kind of randomized controlled interventional study, ANCOVA can answer the following question [45]. “If the groups were equivalent on the pretest, would there be a significant difference between the groups on the posttest?” Thus, ANCOVA should be used in this kind of randomized controlled interventional study [45]. No significant effects were found for any of the psychological measures (*P*>0.05) and morphological data analyzed as described below [*P*>0.05, and corrected at the non-isotropic adjusted cluster level [46] with an underlying voxel level of *P*<0.005]. Thus, since we could not find evidence of a difference between the change of the placebo group and that of the no-intervention group, these two control groups were combined in all subsequent analyses as was performed in a previous WM training study [47]. After that, in another set of ANCOVAs with the same variables, the IATWMMC group, was compared with the combined control group.

In the group level imaging analysis, we tested for group wise differences in the change in rGMV. We used a factorial design option in SPM5. In these analyses, the effects of the interventions, estimated by comparing changes in pre- to post- measures as described above, were compared between the groups at each voxel with total gray matter volume in the pre-measurement, pretest scores of measures of general intelligence (RAPM), and two measures of WM (the arithmetic task of WAIS-III and the letter span task), as covariates. Two measures of WM were included in covariates to rule out the possibility that pre-existing differences in WM affected the extent of WM-training-induced change in rGMV. In the analysis, images representing the changes of rGMV (computed as described above) were compared between groups. Also in the group level imaging analysis, after it was confirmed that there were no significant differences between the effects of placebo training and the effects of no-intervention on rGMV, the two control groups were combined. Then the differences between the effects of IATWMMC and those of the combined control groups were investigated.

In this study, the level of statistical significance was set at *P*<0.05, and corrected for multiple comparisons at the whole brain level using the non-isotropic adjusted cluster level [46] with an underlying voxel level of *P*<0.005. Non-isotropic adjusted cluster-size tests can be applied to data known to be non-isotropic (in another words, not uniformly smooth), such as VBM data [46]. Simulation-based validation of this test has been performed [46], and now it is widely used (in the case of the interventional study, see [48]). In this non-isotropic cluster-size test of random field theory, a relatively higher cluster determining thresholds combined with higher smoothing values of more than 6 voxels are recommended [49]. In this non-isotropic cluster-size test, statistical thresholds were determined based on random field theory [46].

Additionally, we performed simple regression analyses in the IATWMMC group, using the difference between pre- and post- test measures of the letter span task (which is a verbal WM task that showed IATWMMC-related improvement after training in the behavioral analysis described below) and mean rGMV changes in the clusters identified as significant in the analysis of the group comparison, to test for possible correlations between rGMV change and performance change.

Furthermore, to show firmly that preexisting group differences in rGMV did not affect the finding in the group level imaging analysis, we extracted mean value of rGMV changes in pre- to post- measures in the significant clusters in the group level whole brain imaging analysis (ANCOVA) described above as well as that mean rGMV values of pre- measure in these significant clusters. Then, we performed the ANOVA to compare group differences of mean changes in pre- to post- measures in the significant clusters in the group level whole brain imaging analysis (the analysis which did not take preexisting rGMV differences between groups into account) and also we performed ANCOVA to compare group differences of mean changes in pre- to post- measures in the significant clusters with mean rGMV values of pre- measure in these significant clusters as a covariate (the analysis which took preexisting rGMV differences between groups into account). Then we compared significance of two results and saw if the results substantially changed when the mean rGMV values of pre- measures in the significant clusters were taken into account.

## Results

### Basic data

**Figure 1:**
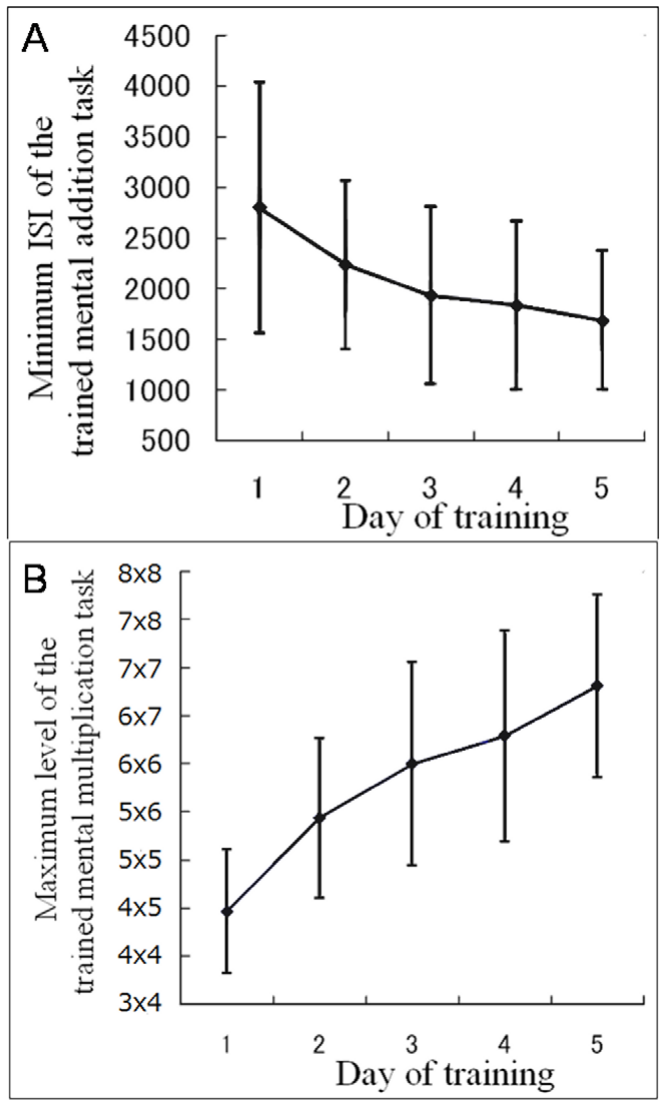
Practice-related performance increase in the group with IATWMMC. Note the individual variation of performance. (A) Practice resulted in a significant increase in trained mental addition task performance (the shortest ISI of the task subjects solved correctly) from the first day of training to the last day of training (paired-t, *P*<0.001). Error bars represent standard deviations. (B) Practice resulted in a significant increase in trained mental multiplication task performance (the highest level of the task subjects solved correctly) from the first day of training to the last day of training (paired-t, *P*<0.001). Error bars represent standard deviations.

All groups were comparable on the relevant background characteristics of age, sex and general intelligence (no significant differences for *P*>0.20, ANOVA). Practice of IATWMMC resulted in a significant increase in trained mental addition task performance (for the shortest ISI of the task solved correctly by the subjects) from the first day of training to the last day of training (paired-t, *P*<0.001; Fig. 1A). Practice-related performance increased in the IATWMMC group. Practice of IATWMMC resulted in a significant increase in trained mental multiplication task performance (the highest level of the task subjects solved correctly) from the first day of training to the last day of training (paired-t, *P*<0.001; Fig. 1B).

### The effect of placebo training on each measure

With regard to the effects of the training on other measures, first the placebo group was compared with the no-intervention group using one-way analyses of covariance (ANCOVAs). The difference between pre- and post- test measures were used as dependent variables and the pretest scores were used as covariates to exclude the possibility that any pre-existing difference in measurement between the groups affected the result of each measure. In all psychological measures, planned statistical tests (one-tail or two-tail) were conducted based on our hypotheses (see Methods for details), as were performed in the previous studies [3], [35]. No significant effects were found for any of the psychological measures (*P*>0.05) or morphological data (*P*>0.05 corrected at non-isotropic adjusted cluster level). Thus, since we could not find any evidence of a difference between the placebo group and the no-intervention group, these two control groups were combined in all subsequent analyses. This was also performed in the previous WM training study [47]. In another set of ANCOVAs with the same variables, the IATWMMC group was compared with the combined control group.

### The effect of IATWMMC on each measure

Behavioral results comparing the combined control group, and the IATWMMC group showed a significantly larger pre- to post- test increase for performance of a complex arithmetic task (*P* = 0.049), for performance of the letter span task (*P* = 0.002), and for reverse Stroop interference (*P* = 0.008) in the IATWMMC group. The IATWMMC group showed a significantly larger pre- to post- test decrease in creativity test performance (*P* = 0.007) (for all the results of the psychological measures, see Table 1). Also the IATWMMC group showed a statistical trend of increase in the mental rotation task (*P* = 0.064). These significant behavioral results remained significant or showed statistical trends when the analyses were performed without data from the no-intervention group, though unsurprisingly the *P* value increased in some tests (for all the results, see Table 1).

**Table 1:** Performance of pretest and posttest in Psychological Measures (Mean ± SEM).

	IATWMMC		Placebo		No-intervention		Planned contrast	P value^b^	P value^c^
	pre	post	pre	post	pre	post			
RAPM^a^	27.3±1.0	31.3±0.7	29.1±0.9	32.0±0.8	27.9±0.7	31.4±0.6	IATWMMC>controls	0.323	0.363
Arithmetic (WAIS-III,score)	19.1±0.7	21.4±0.6	21.6±0.6	23.2±0.5	19.9±0.6	21.8±0.6	IATWMMC>controls	0.535	0.703
Digit-symbol (WAIS-III,score)	104.1±.1	110.2±4.5	106.4±2.8	114.8±2.0	102.1±2.8	110.6±2.6	IATWMMC>controls	0.798	0.780
Reverse Stroop interference (%)	13.4±2.0	22.2±1.9	15.2±2.0	16.1±1.3	17.7±2.3	19.6±2.8	two-tailed	0.008	0.002
Stroop interference (%)	8.5±1.6	7.5±1.5	7.2±2.0	8.2±1.6	9.7±2.8	10.9±2.7	IATWMMC<controls	0.178	0.326
S-A creativity test (total grade)	24.9±1.4	22.6±0.9	26.9±1.7	27.3±1.5	22.9±1.4	24.3±1.3	two-tailed	0.007	0.012
Simple arithmetic (items)	30.7±1.5	33.6±1.3	33.0±1.4	35.4±0.9	34.5±1.2	34.6±1.2	IATWMMC>controls	0.375	0.772
Complex arithmetic (items)	6.53±0.64	8.47±0.79	7.06±0.54	7.38±0.67	7.92±0.71	8.79±0.87	IATWMMC>controls	0.049	0.063
Letter mental rotation (items)	30.8±2.2	46.4±1.9	39.8±2.0	48.8±2.3	35.2±2.4	46.2±2.6	IATWMMC>controls	0.064	0.169
Letter span (score)	16.1±0.8	21.1±0.8	18.0±1.0	20.5±0.8	16.6±0.9	18.6±0.9	IATWMMC>controls	0.002	0.042
Trail making B-A (s)	18.1±1.6	18.2±1.8	25.3±6.8	18.8±2.1	16.2±1.5	16.7±2.0	IATWMMC<controls	0.619	0.498

a Raven's Advanced Progressive Matrices (Raven et al.,1988).

b.c One-way ANCOVAs with test-retest differences in psychological measures as dependent variables and pretest scores of the psychological measures as covariates (b. IATWMMC v.s. Combined controls; c. IATWMMC v.s. Placebo).

**Figure 2:**
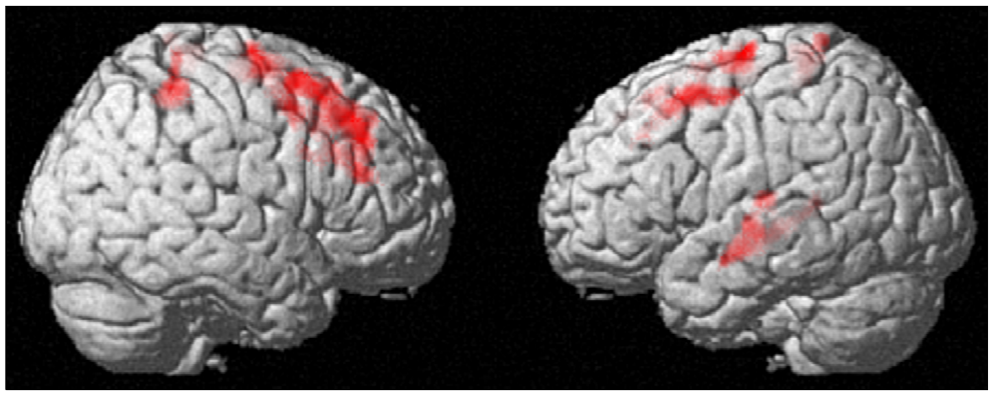
Decrease in gray matter volume in the group with IATWMMC when compared with the combined control group (placebo+no-intervention) (*P*<0.05, corrected for multiple comparisons at the non-isotropic adjusted cluster-level, with an underlying voxel-level of *P*<0.005 uncorrected). Compared with the combined control group, IATWMMC resulted in a decrease in the rGMV of the bilateral DLPFC, bilateral parietal regions and left superior temporal gyrus.

**Figure 3:**
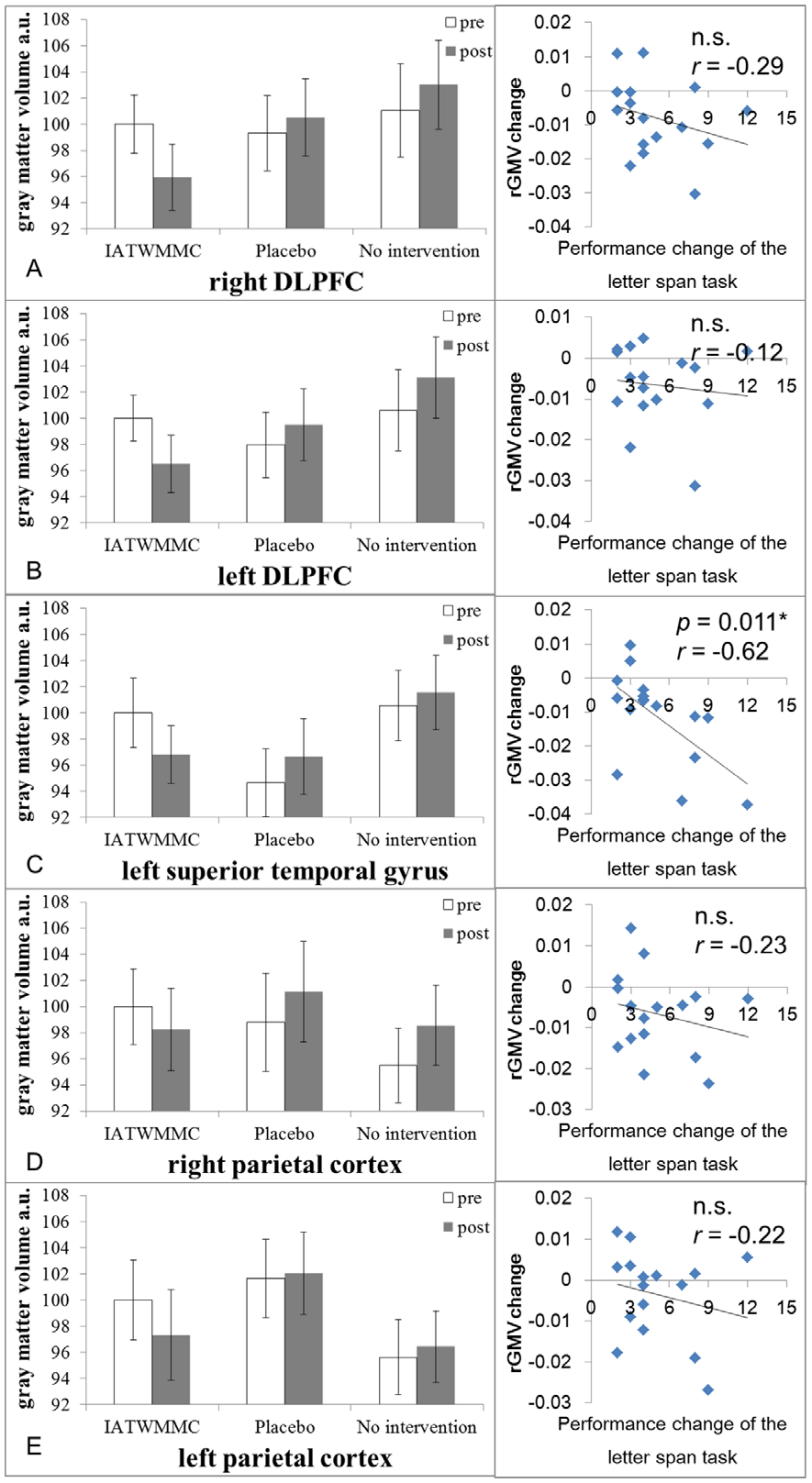
IATWMMC-related changes in rGMV and their associations with the changes in performance in the letter span task. (A, B, C, D, E) Pre- and Post- mean rGMV values in significant clusters in the group with IATWMMC, the group with the placebo intervention, and the group with the no-intervention (left side), as well as scatter plots of the associations between rGMV changes in these clusters and the change in the performance of the letter span task in the IATWMMC group. Bars show mean values for each group. Error bars represent standard errors. The mean rGMV signal value in each cluster was translated so that the mean rGMV signal value in each cluster of the IATWMMC group was 100. Note that there were no statistically meaningful pre-existing group differences in rGMV and the significance of these findings was merely affected when any tendencies of preexisting differences in rGMV were taken in to account (instead of preexisting differences of total brain volume, WMC and general intelligence were taken into account) as shown in the “ANCOVA that takes preexisting rGMV differences in significant clusters into account” section of the Results. Histograms of the mean rGMV value of the significant clusters before and after training for the IATWMMC and control groups, as well as scatter plots, are the following: (A) Right DLPFC. (B) Left DLPFC (two clusters in the left DLPFC were combined). (C) Left superior temporal gyrus in which rGMV changes and changes in performance of the letter span task were significantly correlated. (D) Left parietal cortex. (E) Right parietal cortex.

VBM analysis revealed that, compared with a test-retest decrease in the combined control group, the IATWMMC showed a statistically significantly larger decrease in the rGMV of the bilateral dorsolateral prefrontal cortex (DLPFC), the regions in the bilateral parietal cortices, and the left superior temporal gyrus [(rGM pre - rGM post) IATWMMC - (rGM pre -rGM post) combined control; Fig. 2,3 and Table 2]. There were no significant IATWMMC related increases in rGMV (*P*>0.05, corrected for multiple comparisons at the non-isotropic adjusted cluster level, with an underlying voxel level of *P*<0.005 uncorrected) when compared with the test-retest increase in the combined control groups.

**Table 2:** IATWMMC-related regional gray matter volume changes when compared with the combined control group.

Area		MNI Coordinates			T score	Corrected p value (cluster)
		x	y	z		
(rGMV pre - rGMV post) IATWMMC - (rGMV pre -rGMV post) combined control						
Larger relative decrease in regional gray matter in the group with IATWMMC						
DLPFC	R	33	9	57	4.62	<0.001
Inferior Parietal Lobule	R	47	−43	57	4.47	<0.001
DLPFC	L	−24	−12	71	4.01	<0.001
Paracentral Lobule	L	−4	−37	70	3.98	<0.001
DLPFC	L	−40	7	56	3.79	<0.001
Superior Temporal Gyrus	L	−45	−34	3	3.72	<0.001

### Subjective feelings about the intervention

Using a questionnaire gathered after the training, 10 out of 16 subjects from the IATWMMC group and an almost similar proportion (12 out of 17 subjects) from the placebo training group reported their subjective feelings about the effects of the training (most of the reports were related to task, memory, or mental calculation). The questionnaires also asked each subject about their fatigue during the training using VAS. The mean level of subject fatigue in the IATWMMC group was 7.49 (out of 10) points, while the mean level of subject fatigue in the placebo intervention group was 6.74 (out of 10) points. There were no significant differences in subject fatigue between the two training groups (*P*>0.1). These data, together with our experimental design and the present results, indicate that any subjective feelings of the training effects (or any other factor the placebo intervention group may have had, such as fatigue, commitment to the training, feedback from the training performance, and contact with experimenters) did not lead to an improvement in the performance of the untrained tasks in this kind of study. In our study, monetary reward was given to the subjects of each group in the same way that subjects of noninterventional fMRI studies are recruited; based on how long they participate in the experiment.

### Pre-intervention differences in rGMV

The analysis showed no significant regional differences in rGMV between the IATWMMC group and the combined control group (*P*>0.1, corrected at the nonisotropic adjusted cluster level).

### Regression analysis

Simple regression analyses using the difference between pre- and post- test measures of the letter span task in the IATWMMC group and mean rGMV changes in the clusters that showed the significant effects of IATWMMC revealed there was a significant negative correlation only in a cluster in the left superior temporal gyrus (*P* = 0.011, *t* = −2.94; Fig. 3). The results indicate that the more rGMV decreased in subjects of the IATWMMC group following the training, the more subjects improved on the letter span task.

The lack of correlation in the fronto-parietal clusters (which play a key role in WM) may be because of the nonlinearity of the training induced gray matter change [17], [20]. Also, note number of subjects in the intervention group (N = 16) is apparently not suitable for investigating the number of possible nonlinear relationships. Consistent with this notion, VBM studies have rather consistently failed to identify the linear relationship between gray matter change and intervention-related variables [13], [17], [20], [50].

### ANCOVA that takes preexisting rGMV differences in significant clusters into account

The *P* values of ANOVA (to-tailed) that compare the group differences (between the IATWMMC group and the combined control group) of mean values of pre- to post- changes of rGMV in significant clusters of (a) the right DLPFC, (b) the left DLPFC (the more anterior one), (c) the left parietal cortex (d) the left DLPFC (the more posterior one) (e) the left superior temporal gyrus and (f) the right parietal cortex were 0.0002, 0.0014, 0.0014, 0.0003, 0.00008 and 0.003 respectively. Note this ANOVA did not take preexisting rGMV differences values into account. On the other hand, the *P* values of ANCOVA (two-tailed) that compare the group differences (between the IATWMMC group and the combined control group) of mean values of pre- to post- changes of rGMV with mean values of pre- measure of rGMV in these clusters as a covariate in significant clusters (a)–(f), were 0.0002, 0.0006, 0.0016, 0.0004, 0.00011 and 0.004, respectively (note this analysis is taking preexisting rGMV differences in significant clusters into account). These comparisons show preexisting differences of rGMV in these significant clusters merely affected the group differences of pre- to post- changes of rGMV in these clusters.

When the data from the no-intervention group was removed from the analyses, the same statistical patterns were observed. The *P* values of ANCOVA (two-tailed) that compared the group differences (between the IATWMMC group and the placebo group) of mean values of pre- to post- changes of rGMV with mean values of pre- measure of rGMV in these clusters as a covariate in significant clusters (a)–(f) were 0.007, 0.012, 0.020, 0.004, 0.001, and 0.029, respectively (note that this analysis takes preexisting rGMV differences in significant clusters into account).

## Discussion

The present study revealed the effect of IATWMMC on cognitive functions, and rGMV. Consistent with our hypothesis, IATWMMC changes the brain structure of the bilateral fronto-parietal and the left superior temporal regions, which are critical in WM. Furthermore, IATMMC improved verbal letter span and complex arithmetic performance but it was also associated with an increase in reverse Stroop interference and a decrease in creativity. These changes could not be explained by preexisting differences of each measure between groups. Although, WM (verbal letter span) increase was not associated with changes of the rGMV except in the left superior temporal regions (possibly due to number of reasons including less statistical power (N = 16) and the fact all the subjects went through almost same amount of training in a short period of time and the possibility that there was little meaningful variance among subjects in the IATWMMC group), critically rGMV change was strongly associated with IATWMMC.

A VBM analysis showed, following a five-day IATWMMC, regional GM decreased in the bilateral DLPFC, the regions in the bilateral parietal cortices, and the left superior temporal gyrus, all of which are related to the WM system [1], [51]. Among these regions, the left superior temporal gyrus is consistently activated by language related tasks [52] and plays a key role in the language process. However, this region has also been associated with short-term memory [53] and it is suggested to be a part of the articulatory loop of WM which allows verbal information to be stored in WM [51].

We speculated that one possible mechanism underlying observed structural changes is the usage-dependent selective elimination of synapses [54]. Very rapid experience-dependent structural changes (hours to days after experience) occur continuously at the level of spines and synapses [55]. Selective elimination of synapses helps to sculpt neural circuitry, including that supporting cognitive abilities [56]. Furthermore, a rodent study showed that experience dependent elimination of synapses can happen well within the period of our experiment [57] and together with synaptic formation, it underlies day-to-day experience-dependent neural plasticity [57]. Potential correlates of rGMV include the level of synaptic bulk [12], [58]. Thus, increased synaptic elimination might cause regional GM decreases in this study.

Present results show that cognitive training can cause plasticity in the brain structure of frontal and language related cortices that are presumed to be under strong genetic control based on a noninterventional genetic study [59]. The structure of these regions, especially that of the PFC, is associated with psychometric intelligence [60] and numerous psychiatric diseases (e.g., [61]). Thus, the observed structural changes in the PFC may underlie the observed increased cognitive performance, and the fact that the structure of these regions can change after just a five-day cognitive training may give us new insights into the neural plasticity of these regions and the training's clinical implications [3].

Growing evidence supports that, in certain cases, thinned cortices or cortical thinning are associated with larger or increased cognitive functions (for a review, see [62]). The present findings of a training-related decrease in rGMV occurring with an increase in cognitive functions may have something to do with this phenomenon. A previous developmental study of intelligence showed that superior intelligence is associated with vigorous cortical thinning during adolescence [60]. As a result, the relevant figures showed an inverted U-shaped relationship between cortical thickness in the MPFC and IQ around the age of 20, and the characterization of both superior intelligence and mediocre intelligence by thin cortices. There are a number of other studies which show inverse relationships between regional GM and cognitive functions (for review, see [62], see also [63]). In the other studies, normal cortical development after adolescence is characterized by cortical thinning which occurs most in the frontal lobe during late adolescence and early adulthood [64]. This kind of cortical thinning is one mechanism that underlies the increased efficiency of cognitive processes during skill acquisition [65]. Another previous study showed that cortical thinning was associated with functional activation change in a cohort of older children [66]. Furthermore, while positive correlations between regional GM and cognitive functions have often (though not always) been reported (e.g., see [67] for positive correlations and, [68] for negative correlations) developmental studies of intelligence have shown that children with the highest levels of intelligence show the most vigorous cortical thinning in prefrontal regions during adolescence [60]. The mechanism of developmental cortical thinning, the cross-sectional correlation between regional GM and cognitive functions, and the training-related decrease of rGMV may have a shared and distinct physiological basis. As was already explained, selective elimination of synapses is supposed to underlie both developmental cortical thinning and day-to-day usage-dependent plasticity. As for the cross-sectional correlation between regional GM and certain cognitive functions, it has been speculated that increased developmental cortical thinning is associated with this negative correlation [62], however, it is also possible that the observed usage dependent regional GM decrease (and cognitive improvement) may underlie some of these negative correlations. Yet, these are just speculations and clearly more studies are needed to identify the physiological mechanisms that underlie the increase/decrease in regional brain structures and the positive/negative correlations between regional GM and cognitive functions.

Changes in brain structure after approximately one-week of training or a one-week intervention are consistent with previous studies [17], [18], [19]. However, unlike our study, in these studies there were only increases of regional GM after a one-week intervention [17] or the main changes consisted of increases in regional GM and decreases were minimal or only tendencies [18], [19]. However, critically, another intervention studies of cognitive training in our laboratory using similar training protocols (3–4 h per day, 5 training days in 6 days) both resulted in mainly decrease of rGMV (for one of them, see [69]). Thus, the phenomenon itself is consistent and it is very unlikely that reduction of rGMV after short periods of intense intervention is caused by artifacts or errors. A previous study [17] revealing the time-course of GM change induced by juggling training reported that, weeks after the juggling-training, there were regional GM decreases which followed the initial transient regional GM increases. A similar tendency is observed when older subjects learned to juggle [20]. Our training protocol was short but very intense and concentrated (four hours per day). Thus, one possibility is the observed regional GM decrease happened after the initial increase of regional GM.

IATWMMC not only improves performance of related cognitive tasks such as verbal letter span and complex arithmetic tasks, but it also reduces performance of the creativity task, possibly due to the improved selective attention system following IATWMMC. It has been shown that creativity is positively associated with an impaired selective attention system which does not allow unattended information to be filtered out. Creativity is also associated with psychosis, cognitive disinhibition, or symptoms of attention-deficit hyperactivity disorder (ADHD) [38], [39], [40], [70], [71], [72], [73]. On the other hand, WM training is associated with increased performance for an attention task and the improvement of ADHD symptoms [3], [35], [47]. These results, as well as our findings, are comparable to the study that reported that Ritalin (methylphenidate) administration significantly decreased symptoms of ADHD and creativity [43]. Thus one possible cause of impaired creativity following IATWMMC was a training related improvement in anti-creativity cognitive functions, such as selective attention.

Another interesting result was increased reverse Stroop interference following IATWMMC, which is comparable to decreased reverse Stroop interference caused by aging [36]. However, this result is odd considering the tendency for Stroop interference to decrease following IATWMMC. The exact cause of these data is unknown, especially since the neural correlates of Stroop interference and reverse Stroop interference caused by the matching-type Stroop task (see Methods) (unlike the traditional oral-naming-type) are still largely unknown. Future studies are needed to reveal these issues.

We can exclude the possibility that initial differences in performance between the groups, which might have caused differences in the ceiling effect, led to significant differences in group improvement after training. This is because the pretest scores of each test were added as covariates to the ANCOVA to investigate the difference between the changes in the test performances of each group after training. Furthermore, IATWMMC related changes in performance were not observed for tests in which a few subjects came close to achieving maximum performance. Examples of tests in which a ceiling effect, or maximum performance, could be observed include RAPM and the arithmetic test in WAIS (a measure of WM performance). After training, a few subjects either came close to or actually achieved the maximum performance (36 points and 26 points respectively; while the average performance of posttest of two tests were 31.6 points and 22.1 points respectively) on these test (But note this is in the case of posttest and does not make including scores of these tests in pretest as covariates in VBM analysis problematic). In addition, unlike other tests, these tests might not have been suitable for assessing the effects of a one-week intervention since, in both of these tests, the problems do not consist of countless random and meaningless simple stimuli (letters, digits, colors, symbols and so on). Once subjects know and solve a problem, they may be able to more or less remember the actual problem and solve it very easily after a week's time. Although, the how much subjects remembered the answer or learned how to solve the task are controlled between studies in these tasks as well as any other tests that show learning effects, these problems with the tests might have led to less sensitivity and the negative findings for IATWMMC effects, even though both tests are deeply related to WM (in the case of the Raven test, see [74]).

Looking at these histograms of Fig. 3, it seems there are small (0%<–<2.5%) but consistent increases in rGMV in the control groups in the significant clusters. These increases in the control groups are likely to be caused by two factors. One is statistical deviation in the whole brain imaging analyses and the other is that the control groups tend to show a real increase in rGMV. As for statistical deviations, we performed a whole brain analysis in this study. Even if there are no experimental effects at all, if we perform the same analysis and extract the values of each group in the peak voxels or if we extract the mean values of the insignificant clusters in the analysis, those values will show a tendency of (1) higher rGM value in the pre-measure and lower rGM value in the post-measure in the IATWMMC group (and a resultant rGMV decrease following training) and (2) lower rGM value in the pre-measure and higher rGM value in the post-measure in the control groups (and a resultant rGMV increase following training). If there are no experimental effects, these are just caused by statistical deviations and they happen regardless of whether there are pre training differences in rGM between the groups. The problem is that, even if there are real experimental effects, if we extracted the peak value or mean values of the significant clusters in the whole brain analysis, these values would tend to include the same tendency of statistical deviations described above (though the effects would become relatively weaker) as long as we are dealing with whole brain analyses. This is because the peak voxels of the whole brain analysis are supposedly the voxels where statistical deviations work most to make the values of the voxels match the statistical design (and they are also likely to be the voxels that have strong experimental effects). Furthermore, significant clusters consist of contingent voxels of those peak voxels. Thus, the mean values of clusters have a similar problem. In other words, in these clusters the voxels that did show a 2% decrease in rGMV in the control groups due to sheer statistical deviations are less likely to be included in the significant clusters. As for the real increase in rGMV in the control groups, our other study (Takeuchi et al., unpublished) using data of control groups in one-week intervention studies (including this study) showed a statistically clear rGMV increase in a wide range of areas that overlapped those significant clusters showing an IATWMMC-related decrease in our study, including the right DLPFC, the right parietal cortex, and the left superior temporal gyrus. Furthermore, the increase in rGMV in all of these regions which show increases in rGMV in the control groups were (from strongly to marginally) significantly and positively correlated with an improvement in performance in the cognitive tests (outcome measures). Thus, these increases in rGMV in the control groups may be due to subjects' exposure to the cognitive tests used as outcome measures. The no-intervention group took the outcome measure tests for a wide range of cognitive functions (including working memory) and it took about 3–4 hours to complete these tests. Considering that typical working memory training involves a 10–20 hours training period [8], 3–4 hours of training is not negligible. These tests were not performed with the adaptive procedures known to improve cognitive functions in working memory training [3] (non-adaptive low-level training does not cause any improvement in cognitive functions [3]). However, in most cases, these tests are performed progressively (problems increasingly become difficult and challenge the subjects' limits) or at the most rapid pace (the participants are asked to solve as many problems as quickly as possible in a given time). Either way, these tests are something that challenge the cognitive limitations of the subjects, unlike the placebo training used in this study. In this sense, not only in the behavioral analyses, but also in the rGMV analyses, the no-intervention groups are the groups that show intervention-irrelevant change which should be controlled to see the effect of the experimental intervention on the outcome measures. The increase in rGMV in the control groups is also consistent with the possible mechanisms of rGMV change suggested above (RGMV may decrease after an initial increase based on the training strength and intensity. This is because the 1 day 3–4 hour cognitive tests can be regarded as a mild intervention that does not lead to a decrease in rGMV yet,).

We performed several psychological tests and did not correct for the number of comparisons between statistical tests, as is almost always the case with this kind of study. When corrected using the Bonferroni correction, even after removing the probably void tests (RAPM and WAIS arithmetic), the statistical value for the effect of IATWMMC on the creativity tests marginally surpassed the threshold of *P* = 0.05 (*P* = 0.06). Thus, the results should be interpreted with caution until replicated.

This study has a few limitations that were also common in previous studies of cognitive training (including the most prestigious ones described below). The first limitation is related to multiple (and sometimes heterogeneous) training programs ([3], e.g., [75]) which are, as a general rule, supposed to strengthen transfer effects [76], [77], but may also make it difficult to see the effects of each training program. The second limitation is about the complex training protocols [78], [79], which have none of the strict control groups or conditions which normal fMRI studies have. Mental calculations are typical WM tasks and, as such, they may be suitable for the training of WM, however, they also have numerical components and cognitively complex. Thus, although it would be a statistically challenging work, it would be interesting to disentangle the multiple complex cognitive training protocols and investigate the effect of each component of training in future work. Finally, the training of this study was very brief and long-term effects were not investigated. This is because previous studies have shown one week is long enough to see the effects of cognitive intervention on regional gray matter structures [17], [18], [19] as well as cognitive functions [4], [78] and it is not widely acknowledged that only longer intervention but not 1-week intense intervention does have effects on certain cognitive functions or brain areas, to our knowledge. However, these training protocols make it difficult to compare with several previous WM studies in which training continues for 1–2 months.
